# Supplementary material for: Preventative and therapeutic effects of a GABA transporter 1 inhibitor administered systemically in a mouse model of paclitaxel-induced neuropathic pain
Source: PeerJ. 2016 Dec 15;4:e2798. doi: 10.7717/peerj.2798 (PMC5162398; doi:10.7717/peerj.2798)
Supplement: Supplemental Information 1 [file peerj-04-2798-s001.docx]

**A. Reaction latency times (s) before and taken at day 7 post first administration of paclitaxel or its vehicle in a hot-plate test.**

| **Time after treatment** | **Treatment group** | | | | | | | | | | | | | | | | | | | | | | | | | | | | | |
| --- | --- | --- | --- | --- | --- | --- | --- | --- | --- | --- | --- | --- | --- | --- | --- | --- | --- | --- | --- | --- | --- | --- | --- | --- | --- | --- | --- | --- | --- | --- |
|  | **Vehicle** | | | | | | | | | | | | | | | **Paclitaxel** | | | | | | | | | | | | | | |
| Pretreatment | 10.4 | 10.6 | 8.7 | 9.5 | 10.4 | 9.5 | 10.4 | 10.1 | 9.6 | 10.2 | 10.0 | 9.4 | 9.3 | 10.1 | 9.7 | 9.2 | 11.0 | 9.4 | 9.3 | 9.9 | 10.6 | 9.0 | 10.4 | 10.8 | 9.5 | 8.8 | 9.3 | 9.4 | 9.7 | 8.6 |
| 7 days | 9.0 | 11.3 | 9.8 | 10.6 | 11.1 | 10.6 | 11.1 | 11.1 | 8.7 | 8.2 | 9.9 | 10.2 | 10.2 | 10.1 | 8.0 | 7.7 | 7.7 | 7.7 | 7.0 | 6.9 | 8.3 | 8.4 | 6.4 | 7.0 | 7.9 | 6.8 | 7.8 | 6.5 | 5.9 | 4.3 |

**B.** **Reaction latency times (s) before and taken at day 7 post first administration of paclitaxel or its vehicle in a cold-plate test.**

| **Time after treatment** | **Treatment group** | | | | | | | | | | | | | | | | | | | | | | | | | | | | | |
| --- | --- | --- | --- | --- | --- | --- | --- | --- | --- | --- | --- | --- | --- | --- | --- | --- | --- | --- | --- | --- | --- | --- | --- | --- | --- | --- | --- | --- | --- | --- |
|  | **Vehicle** | | | | | | | | | | | | | | | **Paclitaxel** | | | | | | | | | | | | | | |
| Pretreatment | 34.1 | 60.0 | 55.0 | 53.8 | 50.1 | 47.3 | 60.0 | 47.0 | 60.0 | 52.7 | 60.0 | 60.0 |  |  |  | 60.0 | 58.0 | 55.3 | 60.0 | 52.1 | 52.2 | 60.0 | 31.8 | 60.0 | 54.7 | 60.0 | 60.0 | 60.0 |  |  |
| 7 days | 48.2 | 60.0 | 60.0 | 57.0 | 58.4 | 54.0 | 60.0 | 60.0 | 51.5 | 60.0 | 60.0 | 57.0 |  |  |  | 50.6 | 31.0 | 55.0 | 45.0 | 55.0 | 31.8 | 33.5 | 40.3 | 14.9 | 43.5 | 30.1 | 38.5 | 30.5 |  |  |
