# Supplementary material for: Preventative and therapeutic effects of a GABA transporter 1 inhibitor administered systemically in a mouse model of paclitaxel-induced neuropathic pain
Source: PeerJ. 2016 Dec 15;4:e2798. doi: 10.7717/peerj.2798 (PMC5162398; doi:10.7717/peerj.2798)
Supplement: Supplemental Information 2 [file peerj-04-2798-s002.docx]

|  | **Treatment group** | | | | | | | | | | | | | | | | | | | | | | | |
| --- | --- | --- | --- | --- | --- | --- | --- | --- | --- | --- | --- | --- | --- | --- | --- | --- | --- | --- | --- | --- | --- | --- | --- | --- |
| **Time after treatment** | **Vehicle only** | | | | | | | | **Paclitaxel + vehicle** | | | | | | | | **Paclitaxel + NO-711** | | | | | | | |
| Pretreatment | 10.10 | 9.60 | 10.20 | 10.00 | 9.40 | 9.30 | 10.10 | 9.70 | 9.80 | 9.90 | 10.40 | 9.60 | 9.10 | 11.30 | 9.20 | 9.40 | 9.80 | 8.40 | 9.00 | 9.70 | 9.10 | 8.70 | 9.60 | 9.20 |
| 7 days | 11.10 | 8.70 | 8.20 | 9.90 | 10.20 | 10.20 | 10.10 | 8.00 | 6.00 | 7.10 | 7.10 | 7.00 | 4.50 | 6.10 | 7.00 | 5.70 | 9.80 | 7.20 | 10.00 | 8.90 | 7.40 | 9.20 | 8.90 | 8.40 |

**A. Effects of coadministration of paclitaxel with NO-711 on the development of paclitaxel-induced thermal hyperalgesia in BALB/c mice.**

**B. Effects of coadministration of paclitaxel with NO-711 on the development of paclitaxel-induced cold allodynia in BALB/c mice.**

| **Time after treatment** | **Treatment group** |  |  |  |  |  |  |  |  |  |  |  |  |  |  |  |  |  |  |  |  |  |  |  |
| --- | --- | --- | --- | --- | --- | --- | --- | --- | --- | --- | --- | --- | --- | --- | --- | --- | --- | --- | --- | --- | --- | --- | --- | --- |
| Pretreatment | **Vehicle only** | 60.0 | 59.9 | 47.2 | 60.0 | 60.0 | 60.0 | 60.0 | 60.0 | 60.0 | 60.0 | 60.0 | 60.0 | 60.0 | 58.6 | 52.5 | 60.0 | 60.0 |  |  |  |  |  |  |
| 7 days |  | 55.2 | 51.6 | 37.8 | 60.0 | 30.9 | 31.3 | 60.0 | 32.5 | 60.0 | 60.0 | 58.9 | 60.0 | 55.5 | 60.0 | 60.0 | 58.7 | 54.6 |  |  |  |  |  |  |
| Pretreatment | **Paclitaxel + vehicle** | 60.0 | 60.0 | 60.0 | 60.0 | 60.0 | 60.0 | 60.0 | 60.0 | 60.0 | 60.0 | 58.0 | 57.0 | 58.0 | 60.0 | 60.0 | 57.8 | 55.6 | 52.4 | 54.8 | 60.0 | 55.8 | 60.0 | 54.0 |
| 7 days |  | 58.8 | 41.5 | 44.5 | 24.2 | 21.1 | 14.7 | 36.2 | 31.7 | 35.7 | 32.8 | 32.5 | 25.4 | 40.2 | 38.7 | 33.0 | 20.4 | 30.9 | 18.0 | 30.7 | 31.3 | 30.8 | 35.6 | 27.8 |
| Pretreatment | **Paclitaxel + vehicle** | 60.0 | 60.0 | 60.0 | 60.0 | 58.0 | 60.0 |  |  |  |  |  |  |  |  |  |  |  |  |  |  |  |  |  |
| 7 days |  | 46.8 | 25.66 | 34.2 | 25.0 | 32.5 | 35.3 |  |  |  |  |  |  |  |  |  |  |  |  |  |  |  |  |  |
| Pretreatment | **Paclitaxel + NO-711** | 60.0 | 60.0 | 60.0 | 56.0 | 60.0 | 60.0 | 60.0 | 60.0 | 59.0 | 60.0 | 60.0 | 60.0 | 60.0 | 60.0 | 60.0 | 60.0 | 60.0 |  |  |  |  |  |  |
| 7 days |  | 38.0 | 60.0 | 19.1 | 60.0 | 60.0 | 33.0 | 60.0 | 33.1 | 55.4 | 54.1 | 46.8 | 60.0 | 60.0 | 60.0 | 60.0 | 60.0 | 38.0 |  |  |  |  |  |  |
