# Supplementary material for: Preventative and therapeutic effects of a GABA transporter 1 inhibitor administered systemically in a mouse model of paclitaxel-induced neuropathic pain
Source: PeerJ. 2016 Dec 15;4:e2798. doi: 10.7717/peerj.2798 (PMC5162398; doi:10.7717/peerj.2798)
Supplement: Supplemental Information 3 [file peerj-04-2798-s003.docx]

**A. Reaction latency times (s) (taken at day 7 post first administration of paclitaxel) at different times after treatment with NO-711 (1 and 3 mg/kg) or its vehicle in a hot-plate test.**

| **Time after treatment** | **Treatment group** | | | | | | | | | | | | | | | | | | | | | | | | | | | | | | | | | | | | |
| --- | --- | --- | --- | --- | --- | --- | --- | --- | --- | --- | --- | --- | --- | --- | --- | --- | --- | --- | --- | --- | --- | --- | --- | --- | --- | --- | --- | --- | --- | --- | --- | --- | --- | --- | --- | --- | --- |
|  | **Paclitaxel + vehicle** | | | | | | | | | | | | | | | | | **Paclitaxel + NO-711 1 mg/kg** | | | | | | | | | | | | **Paclitaxel + NO-711 3 mg/kg** | | | | | | | |
| Pretreatment | 9.28 | 7.0 | 10.0 | 7.9 | 7.27 | 7.2 | 7.2 | 6.1 | 7.5 | 7.5 | 6.0 | 6.7 | 7.7 | 7.7 | 7.7 | 7.0 | 6.9 | 7.1 | 5.0 | 6.7 | 7.5 | 6.4 | 7.1 | 7.6 | 7.2 | 8.3 | 8.4 | 6.4 | 7.0 | 5.90 | 4.30 | 7.60 | 7.10 | 6.30 | 6.70 | 6.10 | 6.40 |
| 30 min | 8.06 | 8.0 | 7.0 | 9.6 | 6.8 | 6.1 | 6.1 | 5.16 | 6.2 | 7.8 | 5.9 | 4.1 | 5.7 | 6.9 | 6.9 | 6.1 | 6.6 | 3.4 | 6.6 | 7.2 | 6.5 | 4.6 | 6.1 | 8.0 | 7.0 | 9.3 | 7.1 | 5.9 | 6.2 | 13.00 | 8.00 | 9.80 | 7.90 | 11.10 | 9.10 | 12.00 | 10.00 |
| 1 hr | 9.6 | 8.2 | 8.8 | 7.82 | 6.8 | 5.8 | 6.0 | 5.8 | 7.4 | 7.9 | 5.5 | 4.7 | 6.6 | 5.3 | 7.0 | 7.3 | 6.8 | 4.0 | 5.5 | 7.1 | 7.0 | 4.9 | 7.1 | 8.0 | 7.5 | 9.4 | 7.7 | 5.4 | 6.8 | 10.90 | 8.50 | 10.70 | 8.60 | 9.60 | 7.00 | 7.70 | 7.30 |
| 2 hrs | 9.4 | 10.46 | 7.2 | 10.0 | 7.2 | 6.3 | 5.39 | 5.64 | 7.8 | 8.0 | 5.3 | 3.8 | 6.1 | 7.3 | 6.5 | 7.6 | 6.5 | 3.3 | 6.2 | 6.7 | 6.5 | 5.8 | 6.6 | 8.1 | 7.1 | 10.4 | 7.7 | 6.1 | 6.8 | 9.70 | 7.60 | 7.00 | 6.30 | 8.70 | 7.60 | 7.80 | 7.90 |

**B. Reaction latency times (s) (taken at day 7 post first administration of paclitaxel) at different times after treatment with NO-711 (1 and 3 mg/kg) or its vehicle in a cold-plate test.**

| **Time after treatment** | **Treatment group** | | | | | | | | | | | | | | | | | | | | | | | | | | | | | | | | | | | | |
| --- | --- | --- | --- | --- | --- | --- | --- | --- | --- | --- | --- | --- | --- | --- | --- | --- | --- | --- | --- | --- | --- | --- | --- | --- | --- | --- | --- | --- | --- | --- | --- | --- | --- | --- | --- | --- | --- |
|  | **Paclitaxel + vehicle** | | | | | | | | | | | | | | | | | **Paclitaxel + NO-711 1 mg/kg** | | | | | | | | | | | | **Paclitaxel + NO-711 3 mg/kg** | | | | | | | |
| Pretreatment | 58.00 | 60.00 | 60.00 | 55.60 | 52.40 |  |  |  |  |  |  |  |  |  |  |  |  | 54.00 | 60.00 | 60.00 | 60.00 | 60.00 | 58.00 |  |  |  |  |  |  | 58.00 | 57.00 | 54.80 | 60.00 | 55.80 | 60.00 | 60.00 |  |
| 30 min | 40.20 | 35.70 | 32.80 | 30.90 | 18.00 |  |  |  |  |  |  |  |  |  |  |  |  | 27.80 | 46.80 | 25.66 | 34.20 | 25.00 | 32.50 |  |  |  |  |  |  | 32.50 | 25.40 | 30.70 | 31.30 | 30.80 | 35.60 | 35.30 |  |
| 1 hr | 45.60 | 30.20 | 38.70 | 28.70 | 31.40 |  |  |  |  |  |  |  |  |  |  |  |  | 40.30 | 50.80 | 26.00 | 28.00 | 35.00 | 38.40 |  |  |  |  |  |  | 60.00 | 60.00 | 60.00 | 60.00 | 60.00 | 60.00 | 60.00 |  |
| 2 hrs | 48.80 | 42.80 | 34.40 | 35.70 | 30.80 |  |  |  |  |  |  |  |  |  |  |  |  | 60.00 | 60.00 | 34.00 | 37.50 | 40.30 | 42.00 |  |  |  |  |  |  | 60.00 | 60.00 | 60.00 | 60.00 | 60.00 | 60.00 | 60.00 |  |
