# Supplementary material for: Preventative and therapeutic effects of a GABA transporter 1 inhibitor administered systemically in a mouse model of paclitaxel-induced neuropathic pain
Source: PeerJ. 2016 Dec 15;4:e2798. doi: 10.7717/peerj.2798 (PMC5162398; doi:10.7717/peerj.2798)
Supplement: Supplemental Information 4 [file peerj-04-2798-s004.docx]

|  | **Treatment group** | | | | | | | | | | | | | | | | | | | | | | | |
| --- | --- | --- | --- | --- | --- | --- | --- | --- | --- | --- | --- | --- | --- | --- | --- | --- | --- | --- | --- | --- | --- | --- | --- | --- |
| **Time after treatment** | **Vehicle** | | | | | | | | **NO-711 3 mg/kg** | | | | | | | | **NO-711 5 mg/kg** | | | | | | | |
| Pretreatment | 300 | 300 | 300 | 300 | 300 | 300 | 300 | 300 | 300 | 300 | 300 | 300 | 300 | 300 | 300 | 300 | 300 | 300 | 300 | 300 | 300 | 300 | 300 | 300 |
| 30 min | 300 | 300 | 300 | 300 | 300 | 300 | 300 | 300 | 300 | 300 | 20 | 300 | 300 | 300 | 300 | 300 | 20 | 8.00 | 7 | 300 | 20 | 300 | 32 | 300 |
| 1 hr | 300 | 300 | 300 | 300 | 300 | 300 | 300 | 300 | 300 | 300 | 55 | 300 | 300 | 300 | 300 | 300 | 120 | 300 | 115 | 108 | 180 | 300 | 60 | 300 |
| 2 hrs | 300 | 300 | 300 | 300 | 300 | 300 | 300 | 300 | 300 | 300 | 300 | 300 | 300 | 300 | 300 | 300 | 300 | 300 | 300 | 300 | 300 | 300 | 300 | 300 |

**A. Time course of the mean time spent on the rotarod (s) for two doses of NO-711 (3 and 5 mg/kg) in a rotarod test in naïve BALB/c mice at a constant speed of 4 rpm.**

**B. Time course of the mean time spent on the rotarod (s) for two doses of NO-711 (3 and 5 mg/kg) in a rotarod test in naïve BALB/c mice at an accelerating mode from 4 rpm to 40 rpm over 5 minutes.**

|  | **Treatment group** | | | | | | | | | | | | | | | | | | | | | | | | | |
| --- | --- | --- | --- | --- | --- | --- | --- | --- | --- | --- | --- | --- | --- | --- | --- | --- | --- | --- | --- | --- | --- | --- | --- | --- | --- | --- |
| **Time after treatment** | **Vehicle** | | | | | | | | | | | | | **NO-711 3 mg/kg** | | | | | | | | | | | | |
| Pretreatment | 49.00 | 78.00 | 83.00 | 80.00 | 74.00 | 65.00 | 98.00 | 77.00 | 49.00 | 78.00 | 83.00 | 80.00 | 73.0000 | | 78.00 | 54.00 | 82.00 | 78.00 | 71.00 | 67.0000 | 85.0000 | 74.0000 | 76.0000 | 40.0000 | 56.0000 | 60.0000 |
| 30 min | 85.00 | 61.00 | 62.00 | 92.00 | 82.00 | 60.00 | 49.00 | 99.00 | 85.00 | 61.00 | 62.00 | 92.00 | 5.0012 | | 14.00 | 11.00 | 70.00 | 40.00 | 19.00 | 11.0000 | 16.0000 | 18.0000 | 53.0000 | 7.0000 | 31.0000 | 14.0000 |
| 1 hr | 92.00 | 78.00 | 73.00 | 67.00 | 54.00 | 59.00 | 61.00 | 79.00 | 92.00 | 78.00 | 73.00 | 67.00 | 50.0000 | | 54.00 | 36.00 | 77.00 | 59.00 | 46.00 | 43.0000 | 48.0000 | 46.0000 | 59.0000 | 11.0000 | 43.0000 | 60.0000 |
| 2 hrs | 93.00 | 62.00 | 94.00 | 65.00 | 60.00 | 51.00 | 54.00 | 100.00 | 93.00 | 62.00 | 94.00 | 65.00 | 70.0000 | | 76.00 | 62.00 | 89.00 | 62.00 | 80.00 | 60.0000 | 66.0000 | 53.0000 | 55.0000 | 44.0000 | 59.0000 | 81.0000 |
